# An Evaluation of the Evidence Guiding Adult Midline Ventral Hernia Repair

**DOI:** 10.1055/s-0042-1749428

**Published:** 2022-08-02

**Authors:** Alex Sagar, Niteen Tapuria

**Affiliations:** 1 General Surgery Department, Milton Keynes University Hospital, United Kingdom

**Keywords:** ventral hernia, incisional hernia, laparoscopic hernia repair, robotic hernia repair

## Abstract

**Purpose:** Several guidelines have been published in recent years to guide the clinician in ventral hernia repair. This review distils this advice, critically assesses their evidence base, and proposes avenues for future study.

**Methods:** A PUBMED search identified four guidelines addressing midline ventral hernia repair published by major surgical societies between 2016 and 2020. The studies used to inform the advice have been critically appraised, including 20 systematic reviews/meta-analyses, 10 randomized controlled trials, 32 cohort studies, and 14 case series.

**Results:** Despite a lack of randomized controlled trials, case heterogeneity, and variation in outcome reporting, key themes have emerged.

Preoperative computed tomography scan assesses defect size, loss of domain, and the likely need for component separation. Prehabilitation, frailty assessment, and risk stratification are beneficial in complex cases. Minimally invasive component separation techniques, Botox injection, and progressive pneumoperitoneum represent novel techniques to promote closure of large fascial defects.

Rives-Stoppa sublay mesh repair has become the “gold” standard for open and minimally invasive repairs. Laparoscopic repair promotes early return to functional status. The enhanced-view totally extraperitoneal approach facilitates laparoscopic sublay mesh placement, avoiding mesh contact with viscera. Robotic techniques continue to evolve, although the evidence at present remains immature.

Synthetic mesh is recommended for use in clean and clean-contaminated cases. However, optimism regarding the use of biologic and biosynthetic meshes in the contaminated setting has waned.

**Conclusions:** Surgical techniques in ventral hernia repair have advanced in recent years. High-quality data has struggled to keep pace; rigorous clinical trials are required to support the surgical innovation.

The European Hernia Society (EHS) defines ventral hernias (VHs) as “hernias of the abdominal wall excluding the inguinal area, pelvic area and diaphragm.” 1 2 The EHS classification of VHs is provided in Table 1 .

**Table 1:** Definitions of VHs, set out by EHS 2

Umbilical hernia	Primary VH with its center at the umbilicus
Epigastric hernia	Primary VH close to the midline with its center above the umbilicus
Incisional hernia	VH that developed after surgical trauma to the abdominal wall, including recurrences after repair of primary VHs
Small VH	VH with fascia defect < 1cm
Medium-sized VH	VH with fascia defect 1–4 cm
Large VH	VH with fascia defect >4 cm

Abbreviation: EHS, European Hernia Society; VHs, ventral hernias.

Approximately 2 million VH repairs (VHRs) are performed annually worldwide. 3 In recent years, VH surgery has benefitted from surgical and technological innovation, expanding the limits of what is considered surgically feasible. A wealth of data has been generated over a short period of time. However, the quality of evidence is variable and significant heterogeneity in practice exists. 4

In this context, advancement has been made across the spectrum of VHR, from patient selection and preoperative assessment, through to novel techniques of fascia advancement and minimally invasive repair. Study in this field represents an intersection of biomechanics, material science, and surgery. If a burgeoning relationship between these complementary disciplines can be combined with rigorous clinical trials, we can be cautiously optimistic that the therapeutic possibilities offered to patients will continue to improve.

## Methodology and Limitations of Studies

A PUBMED search was undertaken to identify the guidelines addressing midline (primary and incisional) VHR published by major surgical societies between 2016 and 2020. Guidelines specifically addressing nonmidline VHR were excluded. The following guidelines were included: European Hernia Society (EHS)/American Hernia Society (AHS) 1 ; International Endohernia Society (IEHS) 3 ; Society of Gastro-intestinal Endoscopic Surgeons (SAGES) 5 ; and World Society of Emergency Surgeons (WSES). 6 The key recommendations have been distilled to allow comparison between the guidelines. The review critically appraises the studies used to establish this guidance, identifies areas where evidence is weak, and suggests avenues for future study.

This process has identified a paucity of high-quality data in VHR. Less than 3% of published studies of VHs are randomized-controlled trials (RCTs) 7 ; of the 76 studies in this literature review, 10 are RCTs.

Although primary VHs represent a distinct entity to incisional VHs, 8 analysis is often pooled. Forty-six of the 76 studies in this review combine analysis of primary and incisional VHs. The proliferation of novel surgical approaches and materials in VHR 3 has resulted in a large number of discrete techniques, limiting the total sample size of each and again resulting in pooled analyses of disparate treatments.

The primary outcome measures of most studies (recurrence, patient satisfaction and pain) suffer from lack of standardization in definition 9 10 and measurement. 11 Follow-up tends to be short relative to the usual time-scale of recurrence development. 12

## Patient Selection

Indications for VHR include symptom relief, cosmesis, and avoidance of future emergency presentation. However, nonoperative management in the elective setting is safe. 1

In a cohort study of 1,358 patients with VHs, 636 underwent watchful waiting. The most common reasons for nonoperative management were lack of symptoms, patient comorbidities, and patient's wish. After 5 years, 17% crossover to surgical repair, with 4% presenting emergently. There was no difference in adverse events compared to those who underwent initial operative management. 13

Female gender, advanced age, defect size between 2 and 7 cm, and incisional and umbilical hernias are more likely to incarcerate, supporting elective repair. 14 By contrast, obesity, smoking, and hemoglobin A1c > 6.5% are associated with increased wound morbidity. 15

A RCT of 118 patients with body mass index (BMI) 30 to 40 kg/m ^2^ demonstrated that prehabilitation (nutritional counseling and exercise) resulted in an increase in patients who were complication-free postoperatively. 16 The EHS/AHS guidelines advise weight loss to BMI < 35 kg/m ^2^ and smoking cessation for at least 4 to 6 weeks prior to elective epigastric and umbilical hernia repair. 1

In select cases, this may involve a staged surgical approach: a case series described 15 patients undergoing laparoscopic sleeve gastrectomy followed by staged VHR with favorable outcomes. 17

The Carolinas Equation for Determining Associated Risks (CeDAR) equation was developed through multivariate logistic regression to identify weighted risk factors for wound complications in open VHR, 18 although its reliability has been questioned in other studies. 19

The modified frailty index (mFI) is an additional predictor of complications and mortality following VHR. 20 The factors included in the CeDAR equation and mFI are described in Table 2 . Both CeDAR and mFI provide tools to aid in shared decision-making discussion with patients.

**Table 2:** Features of CeDAR equation and mFI. The OR for surgical site infection in the original CeDAR study are included

CeDAR equation	mFI
Tobacco use (OR: 2.17)	Diabetes mellitus
Previous ventral hernia repair (OR: 2.64)	Partially/totally dependent
Uncontrolled diabetes (OR: 2.01)	COPD/preoperative pneumonia
Presence of stoma (OR: 2.65)	Congestive cardiac failure
BMI > 26 kg/m ^2^ (1.08 per unit BMI)	History of myocardial infarction
Presence of active infection (OR: 2.07)	History of angina/PCI
	Hypertension
	Peripheral vascular disease
	Impaired sensorium
	History of TIA/CVA
	History of CVA with neurological deficit

Abbreviations: BMI, body mass index; CeDAR, Carolinas Equation for Determining Associated Risks; COPD, chronic obstructive pulmonary disease; CVA, cerebrovascular accident; mFI, modified frailty index; OR, odds ratio; PCI, percutaneous coronary intervention; TIA, transient ischemic attack.

## Mesh Selection

Mesh may be synthetic, biosynthetic, or biologic. Of synthetic meshes, medium weight options are associated with fewest complications. 7 Polypropylene is an example of a commonly employed synthetic mesh. In contaminated fields, synthetic mesh carries a prohibitively high surgical site infection rate of 19% 21 and is not recommended. 6 This led to the development of potential alternatives.

Biosynthetic meshes absorb over a period of 6 to 18 months, 22 with the theoretical benefit of reduced surgical site infection. However, this has not been borne out in practice: biosynthetic mesh is associated with increased infective complications compared to biologic and synthetic mesh in clean-contaminated and contaminated surgery, 23 as well as high recurrence rates. 24

Biologic meshes provide a collagen based extracellular matrix scaffold to promote fibroblast collagen deposition, cellular repopulation, and neovascularization. 6 Two types exist: cross-linked and non-crosslinked, with the former being more durable. 25 Although a multicenter retrospective study found biologic mesh in the contaminated setting to be associated a nonsignificant reduction in wound infection and recurrence, 26 this was not confirmed in a systematic review. 27

The LAPSIS RCT assessing mesh use in the clean environment was concluded prematurely due to excessive recurrence rate in the biologic group. 28 A cohort study found biosynthetic mesh to be superior to biologic mesh in elective complex VHR. 29

Biologic mesh is significantly more expensive than synthetic mesh. 30 At present, there is no strong evidence to support its use in contaminated cases. 1 3 7 It is not recommended for large defects in the clean setting. 22 Fundamental studies of biosynthetic and biologic meshes are presented in Table 3 .

**Table 3:** Key studies assessing biosynthetic and biologic mesh outcomes

Reference	Type of study	Sample size	Intervention	Comparison	Follow-up (mo)	Outcome
Sahoo et al 2017 23	Retrospective cohort study	469	Biosynthetic mesh	Synthetic mesh	1	Biosynthetic mesh associated with increased surgical site infection ( *p* = 0.03) and reoperation rates ( *p* = 0.009) compared to synthetic mesh in clean-contaminated and contaminated cases
Renard et al 2020 24	Retrospective cohort study	81	Biosynthetic mesh (Vicryl)	Biologic mesh (Strattice)	36	Biosynthetic mesh associated with increased early ( *p* = 0.03) and late ( *p* = 0.046) infectious complications and recurrence (HR = 0.091 *p* < 0.001) compared to biologic mesh in contaminated incisional hernia repair
Bondre et al 2016 26	Retrospective cohort study	761	Biologic mesh	Synthetic mesh	15	Biologic mesh associated with nonsignificant reduction in infection complication (15.1 vs. 17.8%, *p* = 0.28) and increase in recurrence (17.8 vs. 13.5%, *p* = 0.074) relative to synthetic mesh in contaminated VHR
Lee et al 2014 27	Systematic review	1,304	Biologic mesh	Synthetic mesh	23.2	In clean contaminated cases, biologic mesh associated with increased wound infection rates (31.6%, [14.5–48.7%] vs. 6.4% [3.4–9.4%]) with similar recurrence rates. In contaminated cases, biologic mesh associated with increased recurrence (27.2% (9.5–44.9%) vs. 3.2% (0.0–11.0%) with similar wound infection rates
Miserez et al 2010 28	RCT (prematurely closed)	257	Noncross linked biologic mesh (Surgisis Gold)	Synthetic mesh	12	Biologic mesh associated with higher recurrence across all study arms (laparoscopic 19 vs. 5%; open (11 vs. 3%) in elective VHR
Buell et al 2017 29	Retrospective cohort study	73	Biosynthetic mesh (P4HB)	Biologic mesh (porcine cadaveric)	–	Biosynthetic mesh associated with reduced complication ( *p* < 0.046) and recurrence ( *p* < 0.049) compared to biologic mesh in elective complex abdominal wall reconstruction

Abbreviations: RCT, randomized controlled trial; VHR, ventral hernia repair.

## Plane of Mesh Placement

The keys planes in VH surgery are described in Table 4 . 31 An appreciation of the relevant anatomy is central. 32

**Table 4:** Planes for mesh placement in ventral hernia surgery, adapted from ref. 31

Plane	Anterior relation	Posterior relation
Onlay	Subcutaneous tissue	Anterior rectus sheath and external oblique
Inlay	Mesh attached to edges of hernia defect	
Retrorectus	Rectus abdominis muscle	Posterior rectus sheath
Preperitoneal	Transversalis fascia	Peritoneum
Intraperitoneal	Peritoneum	Abdominal cavity

The EHS/AHS guidelines advise sublay mesh placement for VHR. 1 This refers to mesh placed in either a retrorectus or preperitoneal location. A retrospective cohort study of incisional hernia repairs found sublay placement to improve recurrence and complication rates. 33 The MORPHEUS RCT evaluating primary VHR found preperitoneal mesh to be associated with reduced complications and cost with no difference in recurrence compared to intraperitoneal patch repair. 34 A further cohort study 35 and meta-analysis 36 evaluating both primary and incisional VHs found the retrorectus location to be associated with reduced recurrence and wound infection rates.

By contrast, intraperitoneal mesh placement may promote adhesion formation. In a series of 733 patients undergoing laparoscopic intraperitoneal mesh repair, 2% required reoperation for bowel obstruction after mean follow-up of 19 months. 37

## Antibiotic Prophylaxis

The EHS/AHS, IEHS, and SAGES guidelines advise a single perioperative dose of antibiotics if mesh is used for VHR. 1 3 5 The SAGES guidelines advise cephalosporin (+ vancomycin for patients with known MRSA). 5

A meta-analysis highlighted the paucity of data. 38 The single RCT did not find benefit to antibiotic prophylaxis; however, it included only 19 patients. 39 The guidelines acknowledge that the strength of this recommendation is weak.

## Preoperative Planning and Adjuncts to Abdominal Wall Reconstruction

### Preoperative Imaging

For simple elective primary VHR, the EHS/AHS guidelines recommend that clinical examination should be sufficient. Ultrasound or computed tomography (CT) imaging may be considered if clinical examination is inconclusive. 1

For complex primary and incisional hernias, CT is helpful in preoperative planning 3 : to define defect size, loss of domain (hernia sac volume divided by total peritoneal sac volume 40 ), to predict requirement for component separation, 41 risk of complications, 42 and to guide adjuncts such as preoperative progressive pneumoperitoneum. 40 Loss of domain >15% is likely to lead to significant respiratory impact on return of the visceral contents to the abdominal cavity, 43 while loss of domain >20% is associated with failure of tension-free closure. 44 Visceral fat volume is a significant predictor of recurrence, while hernia sac volume and subcutaneous fat volume predict infection rates. 42

The SAGES guidelines acknowledge the utility of preoperative CT in select cases; however, they reiterate that CT is not able to detect intra-abdominal adhesions or assess abdominal wall compliance, two key factors in operative planning. 5 The IEHS guidelines recommend that dynamic measurement of defect size at different pressures of pneumoperitoneum improves quality of mesh size selection. 3

### Techniques to Allow Fascia Closure

Primary fascia closure (with sublay mesh) is associated with reduced recurrence rates compared to bridged inlay mesh repair. 7 A number of techniques have been developed to extend the abdominal wall musculature to permit this with large defects. The IEHS guidelines advise that these are likely to be required for fascia defects of 8 to 10 cm. 3 Component separation techniques (CSTs) represent the best-studied examples of these methods. 45

Table 5 presents a description of key CSTs.

**Table 5:** Description of component separation techniques

Technique	Description
OACS	Subcutaneous adipose tissue is dissected from the anterior rectus sheath to beyond the linea semilunaris. External oblique is incised along its length and dissected from internal oblique. Rectus abdominis is also separated from the posterior rectus sheath 46
p-OACS	Subcutaneous adipose tissue is dissected from the anterior rectus sheath to beyond linea semilunaris at two distinct sites above and below the umbilicus. These two sites are then joined to create a tunnel over external oblique. The release of external oblique is completed as per the original OACS 47
e-CST	Balloon dissection is used to create a space between external oblique and the subcutaneous adipose tissue. Two further working ports are inserted into this space to incise external oblique and then free it from internal oblique 48
mi-CST	Optical port entry is used to insert a port deep to external oblique. The space between external oblique and internal oblique is developed by carbon dioxide insufflation. Working ports are then inserted and the procedure is completed as per e-CST 49
TAR	Retrorectus space is developed to linea semilunaris. The posterior rectus sheath is incised medial to linea semilunaris to reach transversus abdominis. Transversus abdominis is incised along its length to reach the potential space between transversus fascia posteriorly and transversus abdominis anteriorly. This space is developed laterally 50

Abbreviations: e-CST, endoscopic anterior component separation; mi-CST, minimally invasive anterior component separation; OACS, open anterior component separation; p-OACS, perforator sparing open anterior component separation; TAR, transversus abdominis release.

Open anterior component separation (OACS) allows fascia advancement by approximately 10 cm. However, the undermining of subcutaneous tissue and interruption of perforator vessels leads to up to 40% wound morbidity. 51 This led to the development of alternative techniques. The perforator-sparing OACS spares the periumbilical perforator vessels, with theoretical improvement in wound healing. Endoscopic CST and minimally invasive CST further reduce tissue trauma with intended reduction in wound morbidity.

As with other aspects of VHR, the evidence regarding CSTs is limited by heterogeneity and lack of RCTs. 3 A systematic review found reduced wound complication rates in endoscopic or minimally invasive CST compared to open. 52 Regarding transversus abdominis release (TAR), a meta-analysis reported no difference in wound infection or rate of hernia recurrence between OACS and TAR. 53

Although the IEHS guidelines acknowledge the lack of strong data, they advise consideration of endoscopic/minimally-invasive ACS or TAR as an alternative to OACS to reduce wound morbidity. 3 Importantly, when a CST is used, the associated weakening of the lateral abdominal wall necessitates mesh reinforcement. 3

Additional examples of techniques to improve fascia coverage include preoperative Botox injection, 54 progressive pneumoperitoneum, 55 and tissue expanders. Indeed, Botox injection and progressive pneumoperitoneum can be safely combined to achieve a significant reduction in the ratio of the volume of hernia sac to that of the abdominal cavity. 56 These techniques have been evaluated in a systematic review. 57 All three are safe and may be used in combination with CSTs. However, there is insufficient evidence for them to be recommended at present by the IEHS. 3

In cases where tissue loss will lead to inadequate coverage of the repair, plastic surgical input may be required for split-skin graft or flap reconstruction. 58 In cases with unstable skin coverage, flap closure appears superior to mesh alone. 59 This will require interdisciplinary work with the plastic surgery team.

## Technical Considerations in Open Ventral Hernia Repair

### Primary Ventral Hernia Repair

The EHS/AHS guidelines recommend that mesh should be used for all primary VHRs, regardless of size. 1 A 2018 RCT found reduced recurrence rate when mesh was used to repair umbilical hernias as small as 1 cm. 60 A Danish cohort study also found reduced recurrence rates for primary VHs <2cm when mesh was used. 61 These findings were confirmed in meta-analysis. 62

Subgroup analysis suggests that these findings hold for defects <1cm. 62 However, the EHS/AHS guidelines advise that suture repair alone may be considered for these small hernias. 1 If a suture repair is performed, slowly absorbable or nonabsorbable sutures should be used, 1 although two large Danish population studies found no difference in outcome dependent on suture type. 61 63

The mesh-defect overlap should be 2 cm for defect < 1 cm and 3 cm for defect 1 to 4 cm. 1 However, the data regarding this is conflicting. A systematic review and case series found that for open repairs there was no significant association between degree of overlap and recurrence. 64 65 By contrast, a cohort study found overlap < 1cm to be associated with increased recurrence and in two RCTs (albeit designed to evaluate separate issues), overlap of 3 cm was associated with reduced recurrence. 34 60

There is insufficient evidence to guide a particular technique for mesh fixation, although if the decision is made to fix the mesh, nonabsorbable sutures are advised. With regard to defect closure over the mesh, the guidelines recommend closure although again acknowledge that the evidence is weak. 1

### Incisional Hernia Repair

The higher recurrence rate associated with incisional VHR 8 supports the advice that all incisional hernias should be repaired with mesh. 66 Expert consensus supports sublay repair. 7

## Open versus Laparoscopic Ventral Hernia Repair

The laparoscopic approach should be considered for hernia defects > 4 cm, in addition to patients with defects 1 to 4 cm that are at increased risk of wound infection (e.g., obesity) and for patients with multiple defects. 1 3

The SAGES guidelines advise the factors listed in Table 6 as relative contraindications to the laparoscopic approach. 5

**Table 6:** Relative contraindications to laparoscopic repair of ventral hernia, as per SAGES guidance 5

Significant adhesions
Recurrence hernia
Defect > 10 cm
Unusual location (e.g., subxiphoid, suprapubic)
Loss of domain
Presence of skin graft
Small defect: sac size ratio
Presence of enterocutaneous fistula
Required removal of large mesh

Abbreviations: IEHS, International Endohernia Society; SAGES, Society of Gastro-intestinal Endoscopic Surgeons.

IEHS advises a greater defect size of > 15 cm as a relative contraindication. 3

A Cochrane review 67 demonstrated reduced surgical site infection with the laparoscopic approach, with no difference in recurrence rate. The laparoscopic technique was associated with a higher risk of bowel injury, although this event was rare with a total of 7 enterotomies in 642 cases (5 laparoscopic, 2 open). Limited to primary umbilical hernias, a meta-analysis of 16,549 patients found the laparoscopic approach to be associated with reduced wound infection, recurrence, and length of stay, although longer operating time. 68 The limitations of VHR data discussed in the introduction apply.

An advantage of the laparoscopic technique is that any nearby additional hernia defects are visible at the time of the first operation and can be repaired using the same mesh, avoiding the overlooked additional hernia as a cause of “recurrence.” On the other hand, the lack of an abdominoplasty component with the laparoscopic technique can result in a less favorable cosmetic outcome for larger hernias. The risks of intraperitoneal mesh placement have been described previously.

## Technical Considerations in Laparoscopic Ventral Hernia Repair

The most widely performed laparoscopic technique is the intraperitoneal onlay mesh (IPOM) repair: an intraperitoneal antiadhesion barrier-coated synthetic mesh is placed to cover the defect, recreating the abdominal wall. 1 3

The association between degree of overlap and recurrence is more established for laparoscopic repair than open. Mesh overlap of >5cm was found to be associated with reduced recurrence rate. 64 This approach is advocated by EHS/AHS. 1 A further study found mesh: defect area ratio to be the greatest predictor of recurrence; a mesh: defect ratio of ≥ 16 significantly improves recurrence. 69 The IEHS guidelines recommend this threshold as the determinant of mesh size selection. 3

In addition, the SAGES guidelines highlight that recurrence is reduced where the mesh is fixed lateral to the rectus abdominis. 5 This also reduces the risk of injury to the epigastric vessels.

Various mesh fixation techniques for IPOM exist. The results of key studies are summarized in Table 7 ; no single technique emerges as clearly superior.

**Table 7:** Summary of key studies evaluating different techniques of laparoscopic mesh fixation in ventral hernia repair

Reference	Type of study	Sample size	Intervention	Comparison	Mean follow-up (mo)	Outcome
Reynvoet et al 2014 70	Meta-analysis	4,300	Sutures + tacks	Tacks alone; sutures alone	29.1	No significant difference in recurrence (sutures + tacks 2.5% (1.3–3.7%); tacks 3.4% (2.4–4.5%); sutures 0.9% (0–1.7%) or pain between different techniques
Baker et al 2017 71	Meta-analysis	6,553	Sutures	Absorbable tacks; absorbable tacks + sutures; permanent tacks; permanent tacks + sutures	22	The crude recurrence rates were as follows: absorbable tacks + sutures 0.7%; sutures 1.5%; permanent tacks + sutures 6.0%; permanent tacks 7.7%; absorbable tacks 17.5%. Statistical significance was not achieved in these differences
Brill and Turner 2011 72	Systematic review	8,465	Sutures ± tacks	Sutures alone; tacks alone	30.1	No significant difference in hernia recurrence or prolonged postoperative pain. Sutures associated with significantly higher SSI
Ahmed et al 2018 73	Meta-analysis	466	Tacks	Suture	16.1	No significant difference in postoperative pain at 4–6 weeks (MD: 0.18; 95% CI: −0.48–0.85), chronic pain (OR: 1.24 [0.65–2.38]) or recurrence (OR: 1.11 [0.34–3.62]), although operative time was significantly lower with tack fixation (MD: −19.25 [−27.98–−10.51])
Sajid et al 2013 74	Meta-analysis	207	Tacks	Suture	10.6	No significant difference in recurrence (OR: 1.54 (0.38–6.27). Tacks associated with reduced operative time (MD: −23.65 [−31.06–−16.25]) and 4–6 weeks postoperative pain (MD: −0.69 [−1.16–−0.23])
Khan et al 2018 75	Meta-analysis	1,149	Absorbable tacks	Nonabsorbable tacks	30	No difference in recurrence (RD: 0.03 [−0.04–0.09]) or chronic pain (OR: 0.91 [0.62–1.33])
Eriksen et al 2011 76	RCT	40	Fibrin sealant	Titanium tacks	1	Fibrin sealant associated with reduced acute postoperative pain on days 0–2 ( *p* = 0.025) and resumed normal activity earlier ( *p* = 0.027)
Eriksen et al 2013 77	RCT	40	Fibrin sealant	Titanium tacks	12	Fibrin sealant associated with higher recurrence rates (26 vs. 6%, *p* =0.182), although not statistically significant. No significant difference in pain at 1 year follow-up
Stirler et al 2017 11	Prospective cohort study	80	Absorbable tacks	Titanium tacks	60.5	Early postoperative pain was significantly lower with absorbable tacks at 6 ( *p* = 0.008) and 12 weeks ( *p* = 0.008), but not at 18 months ( *p* = 0.21)

Abbreviations: CI confidence interval; MD, mean difference; OR, odds ratio; RCT, randomized controlled trial; RD, risk difference; SSI, surgical site infection.

The EHS/AHS guidelines advise mesh fixation with either nonabsorbable sutures or tacks. 1 The IEHS guidelines advise either suture fixation or a double-crowned tack technique. 3 The SAGES guidelines do not give specific advice regarding mesh fixation. 5

Similarly, the data regarding the benefit of closing the fascia defect in laparoscopic VHR (a technique termed “IPOM-plus”) is conflicting. These are summarized in Table 8 .

**Table 8:** Summary of key studies evaluating fascia defect closure versus defect nonclosure during laparoscopic ventral hernia repair

Reference	Type of study	Sample size	Intervention	Comparison	Mean follow-up (mo)	Outcome
Nguyen et al 2014 78	Systematic review	393	Defect closure	Defect nonclosure	20	Defect closure results in reduced recurrence (0–5.7 vs. 4.8–16.7%) and seroma rates (5.6–11.4 vs. 4.3–27.8%)
Tandon et al 2016 9	Meta-analysis	3,638	Defect closure	Defect nonclosure	34.8	Defect closure was associated with reduced adverse events (RR: 0.25, *p* < 0.001) and seroma (RR: 0.37, *p* < 0.001)
Gonzalez et al 2014 79	Retrospective cohort study	134	Defect closure	Defect nonclosure	19.4	Defect closure was associated with increased operative time ( *p* = 0.012). There was no significant difference in complications ( *p* = 0.084) or recurrence ( *p* = −0.095)
Lambrecht et al 2015 10	Combined prospective and retrospective cohort study	194	Defect closure	Defect nonclosure	32.5	Defect closure was associated in increased complication rate (OR: 3.42, 95% CI: 1.25–9.33), with no difference in seroma, pain at 2 months, pseudorecurrence or true recurrence

Abbreviations: CI, confidence interval; OR, odds ratio; RR, risk ratio.

The EHS/AHS and IEHS guidelines advise closure of the fascia defect where possible, 1 3 using nonabsorbable sutures. 3 The SAGES guidelines recommend defect closure at the surgeon's discretion. 5

Although the standard laparoscopic technique remains IPOM repair ± fascia defect closure, the potential adhesion-related complications of an intra-peritoneal mesh have prompted EHS/AHS to advocate for sublay mesh placement. 1

Enhanced-view totally extraperitoneal repair 80 is a novel technique that allows laparoscopic preperitoneal retromuscular mesh repair. The initial port incision is used to enter the rectus sheath away from the hernia. The retrorectus space is developed using balloon dissection. Working ports are inserted into this space. The left and right retrorectus spaces are joined and the dissection is continued toward the hernia sac. Sharp dissection is used to drop the hernia sac into the abdomen. The fascia defect is closed and a mesh placed in the dissected retrorectus space. A case series of 79 patients demonstrated the feasibility of this approach with one recurrence after mean follow-up of 332 days. 80 A second case series of 11 procedures demonstrated that this approach can favor the placement of large meshes with no major complication or recurrence after 7 months. 81 However, the data is not yet sufficient to be able to draw firm conclusions. 1

## Robotic Ventral Hernia Repair

Although preperitoneal mesh placement is achievable laparoscopically, this may be facilitated using robotic assistance. 1 3 79 Several robotic VHR techniques exist ( Table 9 ).

**Table 9:** Novel robotic VHR techniques and their more traditional equivalents 3

Robotic technique	Equivalent open/laparoscopic technique
Robotic IPOM	Laparoscopic intraperitoneal onlay mesh repair
Robotic TAPP	Laparoscopic transabdominal preperitoneal mesh repair
Robotic VHR ± robotic TAR	Open retrorectus mesh repair ± transversus abdominis release

Abbreviations: IPOM, intraperitoneal onlay mesh; TAPP, transabdominal preperitoneal; TAR, transversus abdominis release; VHR, ventral hernia repair.

The majority of the evidence regarding robotic VHR derives from case series. No studies to date have sufficient size or follow-up to accurately assess recurrence rates, long-term complications, or to suggest the superiority of one technique over another. 1 3 However, the methods appear promising. 3 Important studies assessing robotic VHR techniques are described in Table 10 .

**Table 10:** Summary of key studies of robotic VHR

Reference	Type of study	Sample size	Intervention	Comparison	Outcome
Gonzalez et al 2014 79	Retrospective cohort study	134	Robotic IPOM-plus	Laparoscopic IPOM	Robotic IPOM-plus associated with nonsignificant reduction in recurrence ( *p* = 0.095) and complications ( *p* = 0.084), with a significant increase in operative team ( *p* = 0.012) compared to laparoscopic IPOM
Kennedy et al 2018 82	Retrospective cohort study	63	Robotic TAPP	Robotic IPOM	Robotic TAPP associated with reduction in complications without significant difference in operative time compared to robotic IPOM
Carbonell et al 2018 83	Retrospective cohort study	333	Robotic RVHR	Open RVHR	Robotic RVHR associated with reduced length of stay ( *p* < 0.001), although with a greater rate of surgical site occurrences (mainly seromas) ( *p* < 0.001) compared to open repair
Bittner et al 2017 84	Retrospective cohort study	102	Robotic TAR	Open TAR	Robotic TAR associated with significant reduction in length of stay (6 days (5.9–8.3 vs. 3 days [3.2–4.3]) but increased operative time ( *p* < 0.01) compared to open TAR

Abbreviations: IPOM, intraperitoneal onlay mesh; RVHR, retromuscular ventral hernia repair; TAPP, transabdominal preperitoneal; TAR, transversus abdominis release.

A limitation with robotic surgery is cost. 85 The cost of equipment (initial purchase and maintenance/disposables) often exceeds $2 million. 3 Further studies into the long-term implications of robotic surgery are required to facilitate cost–benefit analysis. 3

## Management of Emergent and Contaminated Cases

The EHS/AHS and WSES guidelines advise that synthetic mesh repair should be used for incarcerated VHs without strangulation. 1 6 In this setting, an RCT comparing mesh to suture repair for incarcerated paraumbilical hernias demonstrated that mesh was associated with reduced recurrence with no increase in wound infection. 86

In cases of intestinal ischemia without necrosis and bowel resection without gross enteric spillage, synthetic mesh repair can be performed without an increase in wound morbidity. 6 The EHS/AHS guidelines state that this decision should be taken on a case-by-case basis. 1 Although not unanimous, the main studies in this field support the safety of synthetic mesh in this environment ( Table 11 ).

**Table 11:** Key studies assessing use of mesh in emergency VHR (excluding contaminated cases)

Reference	Type of study	Sample size	Intervention	Comparison	Outcome
Haskins et al 2016 87	Retrospective cohort study	2,449, emergency VHR	Mesh repair	Suture repair	Mesh repair was not associated with increased wound-related or additional 30-day morbidity or mortality
Nieuwenhuizen et al 2011 88	Retrospective cohort study	203, emergency groin and VHRs	Mesh repair	Suture repair	Mesh repair was not associated with increased wound complications relative to suture repair
Choi et al 2012 89	Retrospective cohort study	33,832, clean-contaminated and contaminated VHR (elective and emergency)	Mesh repair	Suture repair	Mesh repair was associated with increased complications relative to nonmesh repair in clean-contaminated cases (OR: 3.56 vs. 2.52)

Abbreviations: OR, odds ratio; VHR, ventral hernia repair.

For the stable patient with bowel necrosis or gross enteric spillage during bowel resection, if the defect is < 3cm suture repair is advised. If the defect is too large for suture repair, WSES guidelines suggest consideration of biologic mesh if available. If not, biosynthetic mesh or planned delayed hernia repair are both viable options. 6 However, the evidence for use of biologic and biosynthetic mesh is weak, as described previously; this recommendation remains controversial.

For the unstable patient, open wound management is advised to avoid abdominal compartment syndrome, with early defect closure following stabilization. 6

A number of studies have demonstrated the feasibility of laparoscopy in the management of incarcerated VHs. 90 91 92 A further study extended this to the strangulated setting for groin hernias 93 ; reduced wound infection rates were found in the laparoscopic group without an increase in recurrence. In the emergency setting, the WSES guidelines recommend that laparoscopy may be considered to treat an incarcerated hernia. However, if strangulation or the need for bowel resection is anticipated, the open approach is preferable. 6

A further indication for laparoscopy in the emergency setting is to assess the viability of spontaneously reduced bowel during open repair via hernia sac laparoscopy. An RCT of 95 patients with inguinal hernias found hernioscopy reduced hospital stay and major complications. 94 This could be extended to VHs.

## Conclusion

Although there has been a recent increase in research into VHR, 4 there remain a number of issues that require well-designed RCTs to resolve. These include:

- Comparison of efficacy and safety of different CSTs and tissue expansion techniques.
- Determination of optimal mesh fixation technique in laparoscopic VHR.
- Assessment of benefit of fascial defect closure in laparoscopic VHR.
- Comparison of novel laparoscopic and robotic techniques to standard IPOM.
- Assessment of biologic mesh versus suture repair in contaminated cases.

In addition to these trial topics, improvement in preoperative risk stratification and imaging assessment will improve patient selection.

This review highlights the complexity of VHR; novel techniques and materials develop rapidly, while supporting data struggles to keep pace. The available evidence to guide decision-making is often conflicting and relatively weak. Guidelines must rely heavily on expert consensus.

In this context, challenging cases benefit from discussion in a multidisciplinary setting including radiological, anesthetic, and surgical (both general and plastic surgery) teams. Discussion should focus on consideration of preoptimization, probability of postoperative respiratory impairment, the need for adjuncts to improve fascia coverage, and optimal surgical approach. Careful assessment in this environment helps to bridge the gap between currently available evidence and high-quality patient treatment.
